# Predictors of health care utilization in patients with post-acute sequelae of COVID-19 (PASC)

**DOI:** 10.1371/journal.pone.0331370

**Published:** 2025-09-09

**Authors:** Edward J. Durant, E. Margaret Warton, Jacek Skarbinski, Marcos H. Siqueiros, S. Madhavi Cholleti, David R. Vinson, Dustin G. Mark, Daniel D. DiLena, Adina S. Rauchwerger, Mary E. Reed, Dustin W. Ballard

**Affiliations:** 1 Kaiser Permanente Bernard J. Tyson School of Medicine, Pasadena, California, United States of America; 2 The Permanente Medical Group, Pleasanton, California, United States of America; 3 Kaiser Permanente Clinical Research on Emergency Services and Treatment (CREST) Network, Pleasanton, California, United States of America; 4 Kaiser Permanente Northern California Division of Research, Pleasanton, California, United States of America; UN Mehta Institute of Cardiology and Research Center, INDIA

## Abstract

**Background:**

Research on Post-acute sequelae of COVID (PASC) has focused on the prevalence of symptoms, leaving gaps in our understanding of predictors of health care seeking.

**Objective:**

To identify clinical and sociodemographic characteristics associated with PASC care seeking.

**Methods:**

Retrospective cohort study of adult patients with COVID-19 diagnosis between January 1, 2021 and June 30, 2022 in a community-based comprehensive health care delivery system at 21 hospitals and medical clinics in Northern California. Primary outcome was one or more PASC care seeking encounters at least 28 days after COVID-19 diagnosis in unadjusted and multivariate analyses.

**Results:**

Of 600,295 surviving COVID patients, 3,797 (0.63%) had PASC care encounters. Female sex (RR 1.29, 95% CI 1.20–1.39), non-Hispanic White race, age 40–49 years (RR 2.35, 95% CI 2.08–2.66), more severe acute COVID illness, including an ED visit (RR 4.41, 95% CI 3.92–4.96), and severe depression (RR 1.69, 95% CI 1.32–2.16) were associated with PASC care. COVID immunization (RR 0.79, 95% CI 0.72–0.85), metformin use among diabetic patients (RR 0.74, 95% CI 0.64–0.84), and diagnosis during Omicron predominance (RR 0.54, 95% CI 0.49–0.60) were associated with lower PASC care.

**Conclusion:**

Higher illness severity, medical comorbidities, and infection during the Delta and pre-Delta periods were associated with PASC care seeking. COVID immunization and metformin were associated with lower PASC care seeking. These findings could be useful in understanding the patterns and burden of care seeking for a new disease entity.

## Background

Observational data describe a constellation of signs, symptoms, and conditions that are present four or more weeks after an initial COVID-19 or SARS-CoV-2 infection [1–4]. Commonly referred to as “long COVID,” these have been captured under the umbrella of Post-acute sequelae of SARS-CoV-2 Infection (PASC) to better understand the physiological mechanisms and epidemiology [5]. Efforts are underway to identify the predictors and natural history of PASC as well as the burden of PASC on individual patients and populations [6]. There is less evidence, however, describing the effect of PASC on healthcare utilization. Much of the evidence regarding PASC has focused on the prevalence of PASC symptoms, leaving gaps in our understanding of predictors of healthcare-seeking behaviors among PASC patients.

Measuring the care-seeking impact of PASC symptoms has been challenging as patients and providers may not have been aware of this novel entity in the wake of the early pandemic and a specific diagnosis code for capturing visits did not exist until October 2021. Now we understand that the condition can present across multiple clinical domains, including pulmonary, cardiac, neuropsychiatric, endocrine, and musculoskeletal [7]. It is unclear at which point the burden of PASC translates to additional healthcare utilization and which subset of patients within the PASC cohort are likely to seek medical care for their symptoms. New models of coordinated care, including PASC-specific clinics, have been developed in various clinical settings and healthcare systems for patients requiring ongoing post-COVID care [8]. As a result of the COVID-19 public health emergency, the Centers for Disease Control and Prevention’s National Center for Health Statistics (CDC/NCHS) implemented an additional diagnostic code: U09.9 - post COVID-19 condition, unspecified. This code became effective October 1, 2021 to identify conditions following acute COVID-19. It is a secondary diagnosis code added after the specific condition related to COVID-19 is known [9].

The purpose of this study is to identify predictors of PASC care seeking (healthcare visits with a PASC diagnosis by a clinician) among Kaiser Permanente Northern California (KPNC) adult patients with a preceding COVID-19 positive lab or self-reported home antigen test for diagnosis. This retrospective cohort study focused on the incidence, temporal trends, characteristics, and predictors of PASC-related encounters among adults between January 1, 2021, and June 30, 2022. Our aim was to examine the association between patient sociodemographic and clinical characteristics with PASC care seeking. The study focused on PASC diagnoses that occurred at least 28 days after the initial positive lab or diagnosis. Our hypothesis was that higher severity of initial illness, medical comorbidities, absent or incomplete immunization, and infection with one of the earlier SARS-CoV-2 variants (e.g., Delta) would be associated with a subsequent PASC-related visit.

This investigation contributes to the clinical understanding of the longer-term health care utilization implications of adults infected with COVID-19, with a focus on their intersection with community emergency department (ED) and outpatient care. We anticipate that our findings will inform operational adjustments to address the expected volume of PASC patients and their care-seeking needs as they present to primary care, the ED, and specialty clinics.

## Methods

This retrospective cohort study was undertaken across all 21 KPNC hospitals and affiliated medical clinics of an integrated health care delivery system that provides comprehensive medical care for more than 4.4 million members in the Northern California region. Patients are representative of the ethnic and socioeconomic diversity of the surrounding population [10]. KPNC is supported by a comprehensive, integrated electronic health record (EHR) that includes inpatient, outpatient, emergency, pharmacy, laboratory, and imaging data as well as claims data [11]. KPNC is a learning health care system with an applied research agenda [12]. The KPNC Institutional Review Board approved the study and waived informed consent for this data only study.

**Fig 1:**
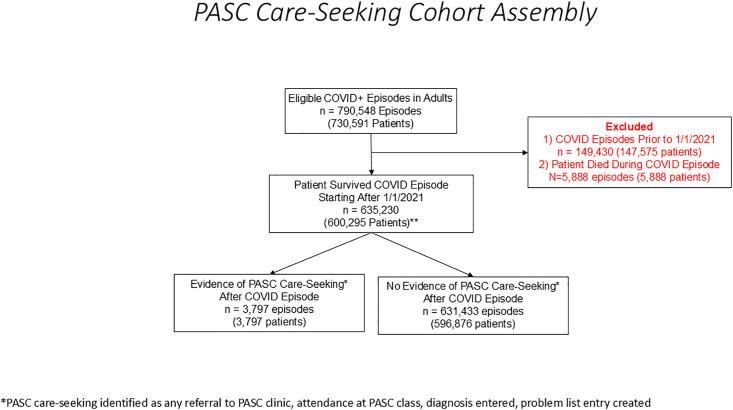
Cohort Assembly.

All KPNC health plan members 18 years or older with a COVID-19 diagnosis (as identified via EHR) between 01/01/2021 and 6/30/2022 were included. A formal ICD-10 diagnosis code for PASC did not exist prior to 01/01/2021. Our complete study cohort included all COVID positive episodes from the beginning of the COVID pandemic, but for this analysis we excluded COVID episodes diagnosed prior to the availability of the PASC ICD-10 code. The Cohort Assembly Diagram (Fig 1) reflects how the complete starting cohort was adjusted and indicates how may COVID episodes were removed for this reason.

The index month was defined as the date of COVID diagnosis for non-hospitalized patients and the date of discharge for hospitalized patients. Patients who died or had fewer than three months of active membership prior to the index month were excluded. The primary study outcome was one or more PASC care seeking encounters that occurred at least 28 days after the initial COVID-19 positive lab test or diagnosis. PASC care seeking was defined as any referral to PASC clinic, attendance at a PASC class, or a new diagnosis or problem list entry of PASC (ICD-10-CM U09.9). Study data were extracted from the EHR for the most recent analysis on 06/07/2023 and last accessed on 06/28/2024. Analysts had access to identifying patient information but it was considered minimal risk and protected as per normal IRB procedures and policies.

In the KPNC system, primary care providers may diagnose PASC in patients who have symptoms for over four weeks not explained by an alternative diagnosis. Patients must have a prior office visit, including a diagnostic workup to rule out alternative causes of symptoms and to address any uncontrolled chronic health conditions. Patients who meet these criteria will have a PASC diagnosis entered on their problem list. Referrals to a PASC consult service are then made by primary care providers and may include a PASC clinic or class, at the referring provider’s discretion. This study included patients who were identified either through the PASC referral system or through a diagnosis code from any clinician.

Exposures of interest extracted from the EHR included demographics (age, sex, and race/ethnicity categories of non-Hispanic White, Black, Asian, Hispanic/Latino, or other), social determinants of health, neighborhood deprivation index (NDI) [13], prior medical history, COVID vaccination status, number of prior COVID-19 episodes, and severity of index COVID illness as indicated by highest level of care (e.g., outpatient, ED, intensive care unit) and respiratory support required (e.g., room air, non-invasive ventilation, intubation). Vaccination status was categorized as no vaccination, incomplete primary series, completed primary series, and completed primary series with at least one booster. We also adjusted for whether this was the patient’s first, second, or subsequent COVID-19 episode. This approach accounted for partial or hybrid immunity to the extent our EHR could capture prior infection history.

Unadjusted bivariate associations were examined using chi-square tests for categorical variables and t-tests with the pooled or Satterthwaite method to determine differences in means of continuous variables. Due to the large cohort size, we also reviewed effect sizes using Cramer’s V for categorical variables and standardized mean differences for continuous variables. Patients could have multiple COVID episodes during the study period, so we assigned any PASC outcome to the most proximate COVID episode that was at least 28 days prior. In multivariate generalized estimating equation Poisson regression models with exchangeable correlation, we adjusted for repeated measures by patient to estimate the relative risk (RR) and 95% confidence intervals (CI) of PASC care seeking behavior within one year.

All available data are presented in the manuscript, tables, figures and supplementary materials in this manuscript. Sharing of additional de-identified data from this study is restricted by the Health Insurance Portability and Accountability Act of 1996 (HIPAA) as a portion of the data is abstracted from electronic health records as part of routine clinical care. De-identified data will be made available upon reasonable request to researchers who meet the criteria for access to confidential data. Please contact kpnc.irb@kp.org.

## Results

Among 790,548 total COVID episodes among 730,591 enrolled patients, we excluded 149,430 COVID episodes starting prior to 1/1/2021 and 5,888 from the final multivariate analysis due to death during index COVID-19 episode. In the surviving cohort, 3,797 patients (0.60%) had 1 or more PASC care seeking episodes (Fig 1). The sociodemographic and clinical characteristics of these patients are shown in Tables 1 and 2, respectively.

**Table 1:** Demographic characteristics of cohort.

	Overall	PASC Care (%)	No PASC Care (%)	P-Value	Effect Size
N	(N = 635,230)	(N = 3797)	(N = 631,433)		
**Age, years**					
Mean (SD)	46.3 (16.5)	50.8 (14.9)	46.3 (16.5)	**<0.001**	0.29
Median (interquartile range)	44.5 (33.3-58.1)	50.2 (39.9-61.2)	44.4 (33.3-58.1)	**----**	**----**
Range	18.0-106.3	18.0-100.3	18.0-106.3	**----**	**----**
Missing, n (%)	0 (0.0)	0 (0.0)	0 (0.0)	**----**	**----**
**Age, category**				**<0.001**	0.03
18-29	112,223 (17.7)	299 (7.9)	111,924 (17.7)		
30-39	143,686 (22.6)	658 (17.3)	143,028 (22.7)		
40-49	129,542 (20.4)	922 (24.3)	128,620 (20.4)		
50-59	110,661 (17.4)	862 (22.7)	109,799 (17.4)		
60-75	105,311 (16.6)	819 (21.6)	104,492 (16.5)		
75 and older	33,807 (5.3)	237 (6.2)	33,570 (5.3)		
**Race/Ethnicity**				**<0.001**	0.02
Black	48,214 (7.6)	350 (9.2)	47,864 (7.6)		
NH White	254,442 (40.1)	1782 (46.9)	252,660 (40.0)		
Asian	121,240 (19.1)	462 (12.2)	120,778 (19.1)		
Hispanic	161,948 (25.5)	957 (25.2)	160,991 (25.5)		
Other/Unknown	49,386 (7.8)	246 (6.5)	49,140 (7.8)		
**Female**	364,870 (57.4)	2491 (65.6)	362,379 (57.4)	**<0.001**	0.01
**Standardized 2019 Neighborhood Deprivation Index**					
Mean (SD)	−0.2 (0.9)	−0.1 (0.9)	−0.2 (0.9)	**<0.001**	0.07
Median (interquartile range)	−0.3 (−0.8-0.3)	−0.3 (−0.7-0.4)	−0.3 (−0.8-0.3)		
Range	−2.4-4.2	−1.9-4.1	−2.4-4.2		
Missing, n (%)	365 (0.1)	3 (0.1)	362 (0.1)		
**Insurance coverage at COVID episode**				**<0.001**	0.01
Medicare	43,575 (6.9)	321 (8.5)	43,254 (6.9)		
Medicaid	80,218 (12.6)	567 (14.9)	79,651 (12.6)		
Other	511,437 (80.5)	2909 (76.6)	508,528 (80.5)		

**Table 2:** Clinical characteristics of cohort.

	Overall (%)	PASC Care (%)	No PASC Care (%)	P-Value	Effect Size
N	(N = 635,230)	(N = 3797)	(N = 631,433)		
**BMI Category**				**<0.001**	0.02
< 30 kg/m^2^	306,937 (48.3)	1627 (42.8)	305,310 (48.4)		
30 and higher	221,092 (34.8)	1687 (44.4)	219,405 (34.7)		
Missing	107,201 (16.9)	483 (12.7)	106,718 (16.9)		
**Smoking Status**				**<0.001**	0.01
Never	453,782 (71.4)	2689 (70.8)	451,093 (71.4)		
Current	33,760 (5.3)	149 (3.9)	33,611 (5.3)		
Former	127,909 (20.1)	894 (23.5)	127,015 (20.1)		
Passive/Unknown	19,779 (3.1)	65 (1.7)	19,714 (3.1)		
**Diabetes at COVID episode start date**	73,151 (11.5)	550 (14.5)	72,601 (11.5)	**<0.001**	0.01
**Patient Health Questionnaire (PHQ-9) Scores**					
PHQ-9 score in year before COVID					
Mean (SD)	7.7 (6.3)	9.2 (6.5)	7.6 (6.3)	<0.001	0.24
Median (interquartile range)	6.0 (2.0-12.0)	8.0 (4.0-14.0)	6.0 (2.0-12.0)	**----**	**----**
Range	0.0-27.0	0.0-27.0	0.0-27.0	**----**	**----**
Missing, n (%)	531,724 (83.7)	2915 (76.8)	528,809 (83.7)	**----**	**----**
PHQ-9 depression category in year before COVID				**<0.001**	0.02
None/Minimal/Mild	68,332 (10.8)	501 (13.2)	67,831 (10.7)		
Moderate/Moderate Severe	29,474 (4.6)	306 (8.1)	29,168 (4.6)		
Severe	5700 (0.9)	75 (2.0)	5625 (0.9)		
No Survey	531,724 (83.7)	2915 (76.8)	528,809 (83.7)		
**Number of Active Diagnoses on Problem List Before COVID**				**<0.001**	0.03
None	45,801 (7.2)	110 (2.9)	45,691 (7.2)		
1-2	97,562 (15.4)	287 (7.6)	97,275 (15.4)		
3-6	194,010 (30.5)	931 (24.5)	193,079 (30.6)		
7-11	151,890 (23.9)	971 (25.6)	150,919 (23.9)		
12 or more	145,967 (23.0)	1498 (39.5)	144,469 (22.9)		
**Disease Severity**					
Highest level of COVID care				<0.001	0.08
None	219,522 (34.6)	578 (15.2)	218,944 (34.7)		
Virtual	300,947 (47.4)	1503 (39.6)	299,444 (47.4)		
Ambulatory	54,174 (8.5)	396 (10.4)	53,778 (8.5)		
Emergency Department	41,802 (6.6)	601 (15.8)	41,201 (6.5)		
Inpatient	14,364 (2.3)	525 (13.8)	13,839 (2.2)		
Inpatient with ICU	4421 (0.7)	194 (5.1)	4227 (0.7)		
Highest level of ventilation during COVID episode				**<0.001**	0.09
None	583,355 (91.8)	2579 (67.9)	580,776 (92.0)		
Room air	37,030 (5.8)	540 (14.2)	36,490 (5.8)		
Nasal cannula	8375 (1.3)	279 (7.3)	8096 (1.3)		
Mask	1664 (0.3)	45 (1.2)	1619 (0.3)		
High-flow nasal cannula	2201 (0.3)	183 (4.8)	2018 (0.3)		
Non-invasive ventilation	1828 (0.3)	107 (2.8)	1721 (0.3)		
Invasive ventilation	777 (0.1)	64 (1.7)	713 (0.1)		
**Therapeutics**					
Vaccination status before COVID				**<0.001**	0.03
None	197,634 (31.1)	1769 (46.6)	195,865 (31.0)		
Primary no booster	213,415 (33.6)	998 (26.3)	212,417 (33.6)		
Primary booster	199,824 (31.5)	878 (23.1)	198,946 (31.5)		
Incomplete	24,357 (3.8)	152 (4.0)	24,205 (3.8)		
On metformin at COVID episode start	37,491 (5.9)	251 (6.6)	37,240 (5.9)	0.06	0.00
**Prevalent Variant at COVID Episode Start**				**<0.001**	0.03
Pre-Delta	87,300 (13.7)	856 (22.5)	86,444 (13.7)		
Delta	115,439 (18.2)	1115 (29.4)	114,324 (18.1)		
Omicron	432,491 (68.1)	1826 (48.1)	430,665 (68.2)		

Table 1 presents completely unadjusted comparisons between groups, using Student’s t-Tests and chi-square tests to test for significant differences and Cramer’s V for categorical and standardized mean differences for continuous measures to estimate effect sizes. Table 2 presents the results from unadjusted generalized estimating equation models for each of the parameters included in the final, fully adjusted model. Note that these unadjusted models do account for clustering by patient.

After multivariate adjustment (Table 3), the risk of PASC care seeking was highest in the age categories of 40–49 and 50–59 years (RR compared with age 18–29: 2.35, 95% CI 2.08–2.66 and 2.23, CI 1.96–2.53, respectively) and lowest in the youngest and oldest age categories (18–29 and greater than 80 years, respectively). Among other demographic variables, non-Hispanic White race or ethnicity and female gender were associated with PASC care seeking, but NDI was not.

**Table 3:** Unadjusted and multivariate adjusted associations between patient socio-demographic and clinical characteristics with PASC care-seeking.

	Unadjusted RR (95% CI)	Multivariate RR (95% CI)	P-value (multivariate)
***Demographics***			
**Age, category (years)**			
Reference: 18–29	1.00	1.00	**----**
30-39	1.72 (1.52, 1.94)	1.66 (1.47, 1.88)	<.0001
40-49	2.54 (2.26, 2.86)	2.35 (2.08, 2.65)	<.0001
50-59	2.77 (2.45, 3.11)	2.23 (1.96, 2.53)	<.0001
60-69	2.72 (2.39, 3.09)	1.85 (1.61, 2.14)	<.0001
70-79	2.58 (2.20, 3.01)	1.36 (1.14, 1.62)	0.001
>=80	2.33 (1.86, 2.91)	**0.87 (0.68, 1.11)**	**0.268**
**Race**			
Reference: non-Hispanic White	1.00	1.00	**----**
Black	**1.04 (0.93, 1.16)**	0.84 (0.74, 0.94)	0.003
Asian	0.54 (0.49, 0.60)	0.70 (0.63, 0.78)	<.0001
Hispanic/Latino	0.84 (0.78, 0.91)	0.86 (0.79, 0.94)	0.000
Other	0.71 (0.62, 0.81)	**0.90 (0.78, 1.03)**	**0.113**
**Sex**			
Reference: male	1.00	1.00	**----**
Female	1.41 (1.32, 1.51)	1.29 (1.20, 1.39)	<.0001
**Standardized Neighborhood** **Deprivation Index**	1.09 (1.05, 1.12)	**0.99 (0.95, 1.02)**	**0.447**
***Patient Health Status***			
**BMI Category**			
Reference: < 30 kg/m^2^	1.00	1.00	**----**
30 and higher	1.44 (1.35, 1.54)	**1.06 (0.99, 1.14)**	**0.113**
Missing	0.85 (0.77, 0.94)	0.73 (0.65, 0.81)	<.0001
**Smoking Status**			
Reference: never	1.00	1.00	**----**
Current	0.75 (0.63, 0.88)	0.66 (0.56, 0.78)	<.0001
Former	1.18 (1.09, 1.27)	0.92 (0.85, 0.99)	0.034
Passive/Unknown	0.56 (0.43, 0.71)	**1.07 (0.82, 1.40)**	**0.605**
**Diabetes and Metformin Status**			
Reference: no diabetes, no metformin	1.00	1.00	**----**
Diabetes and no metformin	1.42 (1.26, 1.59)	0.74 (0.66, 0.84)	<.0001
No diabetes and current metformin	**0.94 (0.55, 1.58)**	**0.64 (0.38, 1.08)**	**0.098**
Diabetes and current metformin	1.18 (1.03, 1.34)	0.74 (0.64, 0.84)	<.0001
**Number of Active** **Diagnoses on Problem List**			
Reference: 0 (no problem list entries)	1.00	1.00	**----**
1-2	**1.23 (0.98, 1.53)**	**1.13 (0.90, 1.42)**	**0.295**
3-6	2.00 (1.64, 2.43)	1.57 (1.27, 1.94)	<.0001
7-11	2.66 (2.19, 3.24)	1.81 (1.46, 2.24)	<.0001
>=12	4.27 (3.52, 5.18)	2.55 (2.05, 3.17)	<.0001
**Pre-COVID PHQ-9 Depression Category**			
Reference: < 10 (none/mild)	1.00	1.00	**----**
10–19 (moderate/mod-severe)	1.42 (1.23, 1.63)	1.44 (1.25, 1.66)	<.0001
20–27 (severe)	1.80 (1.41, 2.29)	1.69 (1.32, 2.16)	<.0001
No PHQ-9 score	0.75 (0.68, 0.82)	0.88 (0.80, 0.98)	0.017
***COVID Severity, Vaccination Status, and Dominant Variant***			
**Highest Level of COVID Care Required**			
Reference: No encounters	1.00	1.00	**----**
Virtual encounter only	1.90 (1.72, 2.09)	1.75 (1.59, 1.93)	<.0001
In-person ambulatory	2.78 (2.45, 3.16)	2.34 (2.06, 2.66)	<.0001
Emergency Department	5.46 (4.88, 6.12)	4.41 (3.92, 4.96)	<.0001
Inpatient (no ICU)	13.89 (12.36, 15.61)	9.71 (8.60, 10.97)	<.0001
Inpatient (ICU)	16.68 (14.21, 19.57)	12.08 (10.17, 14.36)	<.0001
**Pre-COVID Immunizations**			
Reference: no immunizations	1.00	1.00	**----**
Complete primary w/o booster(s)	0.52 (0.48, 0.57)	0.78 (0.72, 0.85)	<.0001
Incomplete primary	0.70 (0.59, 0.82)	**0.90 (0.76, 1.06)**	**0.218**
Complete primary w/ booster(s)	0.49 (0.45, 0.53)	0.87 (0.78, 0.96)	0.006
**Variant Prevalent at COVID Episode**			
Reference: pre-Delta period	1.00	1.00	**----**
Delta	**0.99 (0.90, 1.08)**	**0.96 (0.88, 1.06)**	**0.435**
Omicron	0.43 (0.40, 0.47)	0.54 (0.49, 0.60)	<.0001
**COVID Episode Sequence**			
Reference: first episode	1.00	1.00	**----**
Second episode	**0.95 (0.85, 1.07)**	1.16 (1.02, 1.31)	0.021
Third or higher episode	**0.77 (0.54, 1.11)**	**0.95 (0.65, 1.38)**	**0.779**

Patients with more severe COVID illness were significantly more likely to seek care for PASC. Patients who had completed their primary COVID immunization series and those who had also received a booster immunization were less likely to seek PASC care (RR 0.78, 95% CI: 0.72–0.85 and RR 0.87, 95% CI: 0.78–0.96, respectively). Patients who developed COVID during the Omicron-predominant period (after December 19, 2021) were less likely to seek care for PASC than during the Delta and pre-Delta periods [14].

Several medical comorbidities were associated with PASC care seeking. Patients with a greater number of diagnoses (problem list in the medical record) prior to COVID-19 and those with a higher depression screening Patient Health Questionnaire, version 9 (PHQ-9) score, were more likely to seek care for PASC [15]. Our study did not find a statistically significant association, however, between obesity (BMI > 30 kg/m^2^) and PASC care seeking. Compared with patients who did not have diabetes and were not taking metformin, an active metformin prescription at the time of the index COVID-19 diagnosis was also associated with a lower incidence of PASC care seeking among diabetic patients (RR 0.74, 95% CI 0.64–0.84). Among the small subset of patients without diabetes who were taking metformin (7% of the total patients who were taking metformin, representing 0.41% of patients in our study cohort), we did not find a difference in PASC care seeking (RR 0.64, 95% CI: 0.38–1.42). Current and former tobacco users were less likely to seek PASC care (RR 0.66, 95% CI: 0.56–0.78 and 0.91, 95% CI: 0.85–0.99) than patients who had never used tobacco.

## Discussion

In this retrospective cohort study, we identified factors associated with PASC care seeking among adult patients in a large, community-based integrated health care delivery system. This is the largest study to date to examine predictors of PASC care seeking in an integrated community healthcare setting. Overall, patients with higher illness severity, a greater number of medical comorbidities, and those who had COVID during the pre-Omicron period were more likely to seek care for PASC. We specifically accounted for partial vaccination status (i.e., an incomplete primary immunization series) in our analyses but found minimal protective effect in contrast to patients with a completed series. A completed COVID immunization series was associated with a lower adjusted risk of PASC care seeking.

As the understanding of PASC continues to evolve, observational data have estimated the approximate prevalence of PASC between 3–30% of those with prior COVID-19 infection, with more recent data favoring the lower end of that estimate [3,16]. A longitudinal database study of 1,959,982 COVID-19 patients found a 23% prevalence of at least one post-COVID condition at least 30 days after diagnosis, and a more recent prospective observational cohort study of over 9,700 adult patients had similar findings at least 6 months after diagnosis [7,17]. Recent studies have begun to illuminate potential predictors of PASC. In a deep, multiomic, longitudinal investigation, Su et al. identified four PASC risk factors at the time of initial COVID-19 diagnosis: type 2 diabetes, SARS-CoV-2 RNAemia, Epstein-Barr virus viremia, and specific autoantibodies [18]. Furthermore, Xie et al. demonstrated that individuals with COVID-19 are at increased risk of cardiovascular disease beyond 30 days from infection [6]. This risk was evident even among individuals who were not hospitalized during the acute phase of the infection.

The findings in our study show a similar association with PASC care seeking as those in prior studies reporting a positive association between greater illness severity and PASC. A prospective observational cohort study of over 4,000 patients in the United Kingdom found that those who reported a higher severity of illness were more likely to report ongoing symptoms over 28 days after infection [19]. More recent data also suggest that a higher initial SARS-CoV-2 viral load is predictive of PASC [18]. A large retrospective study of over 8,000 also found severe disease (as well as middle age and certain comorbidities) to be associated with PASC diagnosis or PASC care seeking [20]. Additionally, a study using data from a cohort of 138,818 individuals with confirmed SARS-CoV-2 receiving healthcare through the U.S. Department of Veterans Affairs found that the risk of death, hospitalization and PASC remained elevated even 2 years after infection, especially in patients who were hospitalized during the course of their illness [21].

An association between female sex and PASC care seeking, as we found in our study, correlates with a higher rate of PASC among this population reported across several other studies [19,20]. Our study findings regarding PASC care seeking are consistent with those reporting non-Hispanic White patients to have a higher risk of PASC [20]. Similar to prior studies, we also found that patients in middle age (40–49 and 50–59 years) were at highest risk for PASC care seeking and that the association with age followed a bimodal distribution, with the lowest risks at the ends of the age spectrum.

Our study found two notable factors that were negatively associated with PASC care seeking: Omicron-predominant virus variant at the time of infection and completion of a full COVID immunization series. The relatively lower severity of the Omicron strain in comparison to the earlier Delta and pre-Delta variants is well-documented [22]. Even when controlling for disease severity and immunization status, however, the relative risk of PASC care seeking in our study was approximately half of that in the earlier variants. Similarly, our findings suggesting a protective effect of a complete immunization series (with or without boosters) aligns with similar findings in a prospective, community-based, case-control study from the United Kingdom [23]. An incomplete primary immunization series, however, seems to offer minimal protection.

Data vary on medical comorbidities. Depression has been frequently cited as a risk factor for PASC or PASC care seeking, both in large retrospective cohort studies as well as smaller case-control studies [19,20,24]. Although obesity (BMI ≥ 30 kg/m^2^) did not show a statistically-significant association with PASC care seeking in our study or other smaller studies, some of the larger studies have reported it as a risk factor [19,20].

Diabetes, which has previously been reported as a risk factor for PASC in other studies showed a negative association with PASC care seeking in our study [20]. It is unclear whether this was due to collinearity with other variables (such as illness severity), unmeasured confounders, or the attribution of non-specific PASC symptoms to chronic illness rather than to PASC. Notably, among diabetic patients, we observed that metformin use was associated with lower PASC care seeking, suggesting that metformin may partially mediate or confound the relationship between diabetes and PASC. Although a recent study showed a relative decrease in the incidence of PASC in patients taking metformin compared to the blinded control in a randomized phase 3 trial, the number of non-diabetic patients in our study who were taking metformin was likely too small to provide sufficient power to determine whether the same association could be observed with PASC care seeking [25].

Interestingly, our study found a somewhat puzzling negative association between tobacco use and PASC care seeking [20]. Although this finding may seem counterintuitive as the relationship between tobacco use and increased healthcare utilization is well-documented, there is also evidence that tobacco users tend to delay seeking care for certain respiratory symptoms and physical discomfort, which can be features of PASC [26–28]. This, at least, seems a more plausible explanation than a heretofore undiscovered beneficial effect of tobacco use [24].

It is important to note that the proportion of patients seeking care for PASC in our study was small relative to the number of patients expected to have PASC, based on the prevalence of PASC reported in other studies. The patients in this study were those who felt they were sick enough to seek care for PASC, which is a higher threshold and reflects a higher level of concern than merely reporting symptoms in a survey. At least some of the gap between expected prevalence and actual care seeking, however, was likely due to underdiagnosis and misclassification of PASC symptoms.

Our study had several limitations. First, this was a retrospective cohort study using EHR data, thus we cannot rule out unmeasured confounding and the findings should not be interpreted causally. Second, although our data source was a comprehensive EHR, which likely limited data fragmentation (missing data from sources not connected to our healthcare system), EHR data can suffer from missing data, limited variables, and misclassification. Third, we conducted our study at a large, integrated health care system of community hospitals, which may limit generalizability to other settings. Finally, we chose to examine the primary outcome of PASC care seeking as a discrete health care outcome. Our outcome did not capture all patients with PASC symptoms, only the patients who sought medical care for PASC at our institution. Certainly not all patients with PASC seek care and certain symptoms are likely associated with higher care seeking than others. The population of patients with PASC symptoms that did not seek care or sought care outside of our integrated healthcare system also may have differed from the patients in our PASC care-seeking cohort. This may limit generalizability among patients who tend to seek alternative therapies or are generally care-avoidant, including those who are uninsured or underinsured.

## Conclusions

In this retrospective cohort study examining predictors of PASC care seeking among nearly 800,000 patients with COVID-19, we found that higher illness severity, medical comorbidities, and infection during the Delta and pre-Delta periods were associated with PASC care seeking. A completed COVID immunization series and metformin were associated with lower PASC care seeking. Ours is the largest study to date to examine predictors of PASC care seeking in an integrated community healthcare setting. These findings could be useful in understanding the patterns and burden of care-seeking for a new disease entity. This could contribute to future research and lead to a better understanding of these associations and their underlying mechanisms.
